# Gut microbiota characteristics of Mongolian and Han populations in anti-tuberculosis drug-induced liver injury: a population-based case–control study

**DOI:** 10.1186/s12866-023-02801-4

**Published:** 2023-03-16

**Authors:** Jinqi Hao, Yuhong Li, Yanqin Yu, Limin Zheng, Fumin Feng

**Affiliations:** 1 School of Public Health, North China University of Science and Technology, Hebei Province 063210 Tangshan, China; 2 School of Public Health, Baotou Medical College, Inner Mongolia 014030 Baotou, China; 3 The First Affiliated Hospital of Baotou Medical College, Inner Mongolia 014010 Baotou, China

**Keywords:** Pulmonary tuberculosis, Anti-tuberculosis drug-induced liver injury, Gut microbiota imbalance

## Abstract

**Background:**

The pathogenesis of anti-tuberculosis (TB) drug-induced liver injury (ADLI) is complicated and remains unclear. We aimed to analyse the relationship between the characteristics of gut microbiota and ADLI in Mongolian and Han patients with pulmonary TB and identify the most notable bacteria related to the occurrence of liver injury in those populations.

**Methods:**

Patients with concurrent liver injury (LI) and no liver injury (ULI) before receiving first-line anti-TB drug treatment (T1) from the Han population in Tangshan and the Mongolian population in Inner Mongolia were selected as research subjects. At the time of liver injury (T2), stool samples were measured by bacterial 16S rRNA gene high-throughput sequencing to analyse and compare the differences in the gut microbiota of the LI and ULI Mongolian and Han patients at T1 and T2 and identify the differences between those patients.

**Results:**

A total of 45 Mongolian and 37 Han patients were enrolled in our study. A dynamic comparison from T1 to T2 showed that the microbiota of the LI and ULI groups changed significantly from T1 to T2 in both the Mongolian and Han populations. However, there were commonalities and personality changes in the microbiota of the two ethnic groups.

**Conclusion:**

Differences in gut microbes in ADLI were found among the Han and Mongolian patients in our study. *Ekmania* and *Stenotrophomonas* were related to the occurrence of ADLI in Mongolian patients, while Ekmania and *Ruminococcus__gnavus_group* were related to the occurrence of ADLI in the Han population.

## Introduction

Tuberculosis (TB) is one of the chronic infectious diseases that seriously endangers human health, and its morbidity and mortality rank second among the notifiable infectious diseases in China. Currently, short-course directly observed treatment is the most effective strategy for TB control. Anti-TB drug-induced liver injury (ADLI) is part of the most important anti-TB treatment strategy and one of the most serious adverse reactions, accounting for more than 7.0% of total adverse reactions [1]. Due to the combination of multiple first-line anti-TB drugs, the pathogenesis of ADLI is complicated and remains unclear [2]. The incidence of ADLI in China is also relatively high, ranging from 2.5 to 11.9% [3, 4]. It is an important cause of anti-TB treatment failure, recurrence, and drug resistance in patients, which seriously affects the overall effect of TB epidemic control.

Recently, drug-induced hepatotoxicity studies have demonstrated that the gut microbiota can influence drug metabolism, thereby affecting drug efficacy and toxicity [5–7]. Generally, anti-TB drugs are taken orally and are in direct contact with the intestinal microbiota. There is tremendous variation in the therapeutic response of individuals to orally administered drugs. The intestinal microbiota produces many enzymes that have the potential to metabolize drugs. This interplay may activate/inactivate a drug and in some cases, produce toxic compounds. Studies have also shown that anti-TB drugs can lead to an imbalance of intestinal microbiota in patients with pulmonary TB [8, 9]. Although these studies have demonstrated a link between the intestinal microbiota and drug-induced liver injury, the study of intestinal microbiota on drug-induced liver injury is still in its infancy and is only the tip of the iceberg. The mechanisms of many effects are yet to be discovered in the relationship between ADLI and intestinal microbiota.

To prevent the occurrence of severe liver injury, it is particularly urgent to identify the occurrence of liver injury early and provide timely intervention. Thus, to explore the association between faecal microbiota characteristics and the occurrence of ADLI, faecal DNA extraction and 16S rRNA gene data analysis were performed to determine the homogeneity and heterogeneity of intestinal microbial patterns in patients with ADLI from different populations. We also identified shared microbiota markers associated with ADLI occurrence in Han and Mongolian patients.

## Materials and methods

### Research subjects

Convenience sampling was used to select Han patients with pulmonary TB who were hospitalised in Tangshan Fourth Hospital and Mongolian patients with pulmonary TB who were hospitalised in the Hulunbuir Infectious Disease Hospital in Inner Mongolia from October 2017 to October 2018 as research subjects.

Inclusion criteria: (a) patients with newly diagnosed TB according to the ‘WS 288-2017 Diagnosis of Pulmonary Tuberculosis’ standard, (b) aged 18–60 years, (c) with a daily regimen of the standard regimen 2HRZE/4HR (H = isoniazid, R = rifampicin, Z = pyrazinamide, E = ethambutol) during this study, (d) with no use of antibiotics, gastrointestinal motility drugs, or microecological regulators in the past month and (e) all patients were informed, willing to participate independently and could maintain close follow-ups.

The diagnosis of TB was made as follows: (a) moderately or strongly positive in purified protein derivative with TB imaging features, (b) positive in Interferon-γ-release assays with TB imaging features, (c) positive in anti-TB antibody (IgM) with TB imaging features and (d) lung histopathology TB lesion with TB imaging features.

Exclusion criteria: (a) patients with other diseases that were unsuitable for inclusion in this study or directly related to the gut microbiome, such as severe heart, brain, lung, liver and renal system diseases; chronic inflammation, autoimmune diseases, diabetes and gastrointestinal diseases, (b) patients with changes in their condition during anti-TB treatment and those who used antibiotics other than TB drugs and a large number of hormones. This study obtained the informed consent of the patients and their families and was approved by the Ethics Committee of Baotou Medical College (approval no. 2018–002).

**Fig. 1:**
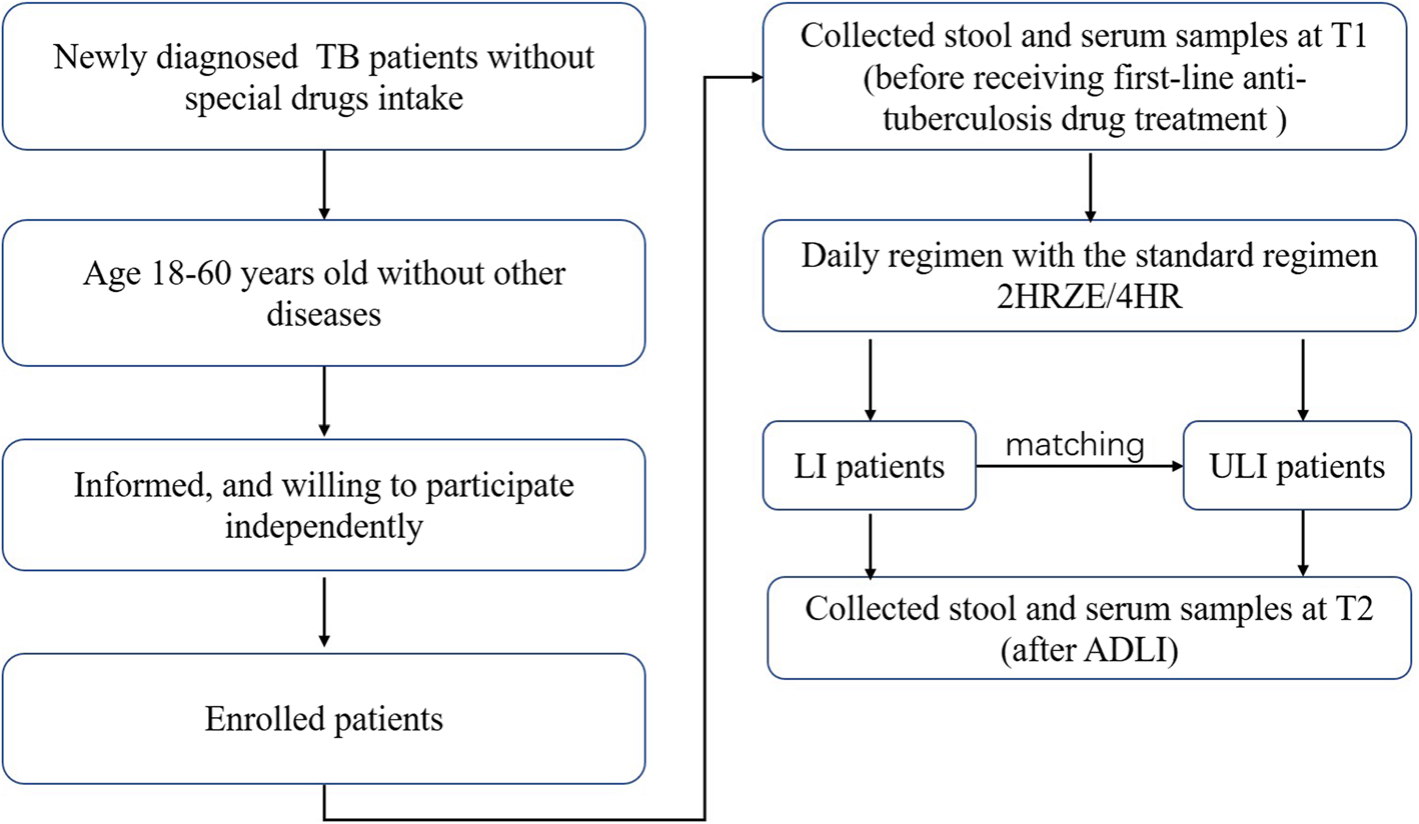
The flowchart of this study design

Based on liver enzyme results according to the criteria of the American Thoracic Society, ADLI was described as follows: (a) an increase of more than threefold in serum alanine transaminase (ALT) or aspartate aminotransferase (AST) compared with the upper limit of normal (ULN) in the presence of symptoms of liver injury; (b) an increase of more than twofold in serum total bilirubin (TBIL) compared with the ULN in the presence of symptoms of liver injury and (c) an increase of more than fivefold in serum ALT, AST and TBIL compared with the ULN in the presence or absence of symptoms of liver injury. The patients who developed liver injury within 3 to 4 weeks after taking anti-TB drugs were selected as the liver injury group (LI), and the patients who received treatment during the same period without liver injury, who were selected according to gender and ethnicity frequency matching, were selected as the no liver injury group (ULI). A summary of the study design is shown in Fig. 1.

### Methods

#### Extraction of total faecal bacterial DNA

Faecal specimens from all subjects were collected before anti-TB drug treatment (T1) and at the time of liver injury (T2). The patient defecated into a sterile stool box in the morning. After sub-packaging and marking, a sterile toothpick was used to extract a stool sample of about 0.2 g from the middle of the stool and place it in a sterilized cryopreservation tube. The extraction of total DNA from the faecal intestinal bacteria of all subjects was performed in strict accordance with the instructions of the QIAamp DNA Stool Mini Kit.

#### Detection of serum liver function indicators

All patient sera were collected before anti-TB drug treatment and after ADLI. In the morning, fasting 5 mL of peripheral venous blood was collected in non-anticoagulant sterile blood collection tubes and left at room temperature before centrifuging at 3000 r/min for 10 min. The supernatant sera were tested by an automatic biochemical analyser to detect the liver function indexes of ALT and AST activity, and alkaline phosphatase (ALP) and TBIL.

#### PCR amplification of the V3–V4 hypervariable region of the 16S rRNA gene

The general primers of the V3-V4 hypervariable region of the 16S rRNA gene were selected for PCR amplification on the extracted total faecal DNA. The primer sequences were supported by a commercial sequencing company with forward primer (5′-NNNNCCTACGGGAGGCAGCAG-3′) and reverse primer (5′-NNNNGGACTACHVGGGTATCTAATCC-3′) [10]. The NNNN added to the 5′ end of the upstream and downstream primers is a 4-bit label specific to each sample. The base sequence can achieve the purpose of simultaneous sequencing of multiple samples.

#### Construction of sequencing library and high-throughput sequencing

We constructed a DNA library in strict accordance with NEB’s (NEBNext® Ultra™ II DNA Library Prep Kit for Illumina®) operating instructions and increased the product to obtain the library. After the library was completed, it was diluted to 1 ng/μL, and the quality of the library was tested according to the instructions of Agilent’s High Sensitivity DNA kit 2100. After confirming that the library was qualified, it was accurately quantified by real-time fluorescent quantitative PCR and sequenced by an Illumina MiSeq system.

#### Sequencing data processing

The paired-end sequence data obtained by MiSeq sequencing was spliced into one sequence by FLASH software (version 1.2.10), and fastp (version 0.19.6) was used to filter the quality of the reads and the effect of the merge. The primer sequences differentiated the samples to obtain valid sequences and corrected the sequence direction to obtain clean reads.

Clustering was performed using USEARCH (version 7.0.1090) at a similarity of 0.97, and after chimera filtering was performed on the clustered sequences, the operational taxonomic unit (OTU) for species classification was obtained. A representative sequence was taken from the same OTU for species annotation analysis, and RDP Classifier (version 11.5) was used to perform species annotation on the representative sequence. Silva (Release132 http://www.arb-silva.de) compared (confidence threshold was set to 70%). The composition of each sample was counted at the taxonomic level of domain, kingdom, phylum, class, order, family, genus, and species. Mothur software (version 1.30.2) was used to analyse the alpha diversity index of the bacterial flora in each group of samples. Phylogenetic-based unweighted and weighted UniFrac distance matrices were calculated using QIIME software (version 1.9.1).

### Observation indicators

Baseline data on general demographic characteristics, lifestyle and behavioural habits, and anti-TB treatment status of the study subjects were collected. Liver function biomarkers, such as liver enzymes and bilirubin, were recorded.

### Statistical analysis

The R language package and SPSS 22.0 software were used to process and analyse the data. Measurement data conforming to and not conforming to a normal distribution were expressed as mean ± standard deviation (± SD) and median (interquartile range) [M (IQR)], respectively. Paired *t* tests and independent-sample *t* tests were used to measure the differences between groups for normally distributed data, and Mann–Whitney–Wilcoxon tests and Mann–Whitney U tests were used to measure differences between groups for non-normally distributed data. The enumeration data were expressed by rate or composition ratio, and the *X*^2^ test was used to measure the difference between the rate or composition ratio. Linear discriminant analysis effect size (LEfSe) was used to analyse differentially enriched taxa in intestinal microbial communities between groups. Any taxonomic group with a linear discriminant analysis (LDA) effect size > 2 and a *P* value < 0.01 or < 0.05 was considered statistically significant. The correlation analysis between intestinal microbiota and clinical biochemical indexes was performed using Spearman’s correlation coefficient analysis.

## Results

### Basic characteristics of the research subjects

A total of 19 Mongolian patients with liver injury and 26 without liver injury, and 17 Han patients with liver injury and 20 without liver injury were included in this study. There were no significant differences in age, gender, body mass index, marital status, place of residence, smoking status and drinking status between the Han patients with and without liver injury (all *P* > 0.05). There was no statistical difference between the two groups (all *P* > 0.05). There were no significant differences in these indicators between the Mongolian and Han populations with liver injury and those without liver injury (all *P* > 0.05) (see Table 1).

**Table 1:** Baseline clinical characteristics of respondents

Index		Han nationality(*n* = 37)			Mongolian(*n* = 45)			*P*^*+*^	*P*^*++*^
		LI(*n* = 17)	ULI(*n* = 20)	*P*	LI(*n* = 19)	ULI(*n* = 26)	*P*		
Age (years)		32.0 (25.0–50.5)	37.5 (26.5–45.5)	0.245	37.0 (26.0–55.0)	30.5 (23.0–52.0)	0.988	0.288	0.947
BMI (kg/m^2^)		19.5 (18.0–21.7)	21.2 (18.8–23.1)	0.340	19.3 (17.9–21.9)	20.2 (18.9–21.8)	0.247	0.739	0.485
Sex	Male	10 (58.8)	14 (70.0)	0.478	11 (57.9)	15 (57.7)	0.615	0.955	0.336
	Female	7 (41.2)	6 (30.0)		8 (42.1)	11 (42.3)			
Marriage	Unmarried	6 (35.3)	6 (30.0)	0.732	4 (21.1)	10 (38.5)	0.179	0.341	0.550
	Married	11 (64.7)	14 (70.0)		15 (78.9)	16 (61.5)			
Place of residence	City	5 (29.4)	7 (35.0)	0.717	6 (31.6)	11 (42.3)	0.338	0.888	0.615
	Countryside	12 (70.6)	13 (65.0)		13 (68.4)	15 (57.7)			
Smoking	No	11 (64.7)	15 (75.0)	0.495	12 (63.2)	19 (73.1)	0..349	0.923	0.883
	Yes	6 (35.3)	5 (25.0)		7 (36.8)	7 (26.9)			
Drinking	No	12 (70.6)	14 (70.0)	0.969	14 (73.7)	18 (69.2)	0.506	0.836	0.955
	Yes	5 (29.4)	6 (30.0)		5 (26.3)	8 (30.8)			

*P* is the comparison between the LI group and the ULI group; *P*^*+*^ is the comparison between the Han LI group and the Mongolian LI group; *P* ^*++*^ is the comparison between the Han ULI group and the Mongolian ULI g

### Analysis of differences in species composition of intestinal microbiota

**Fig. 2:**
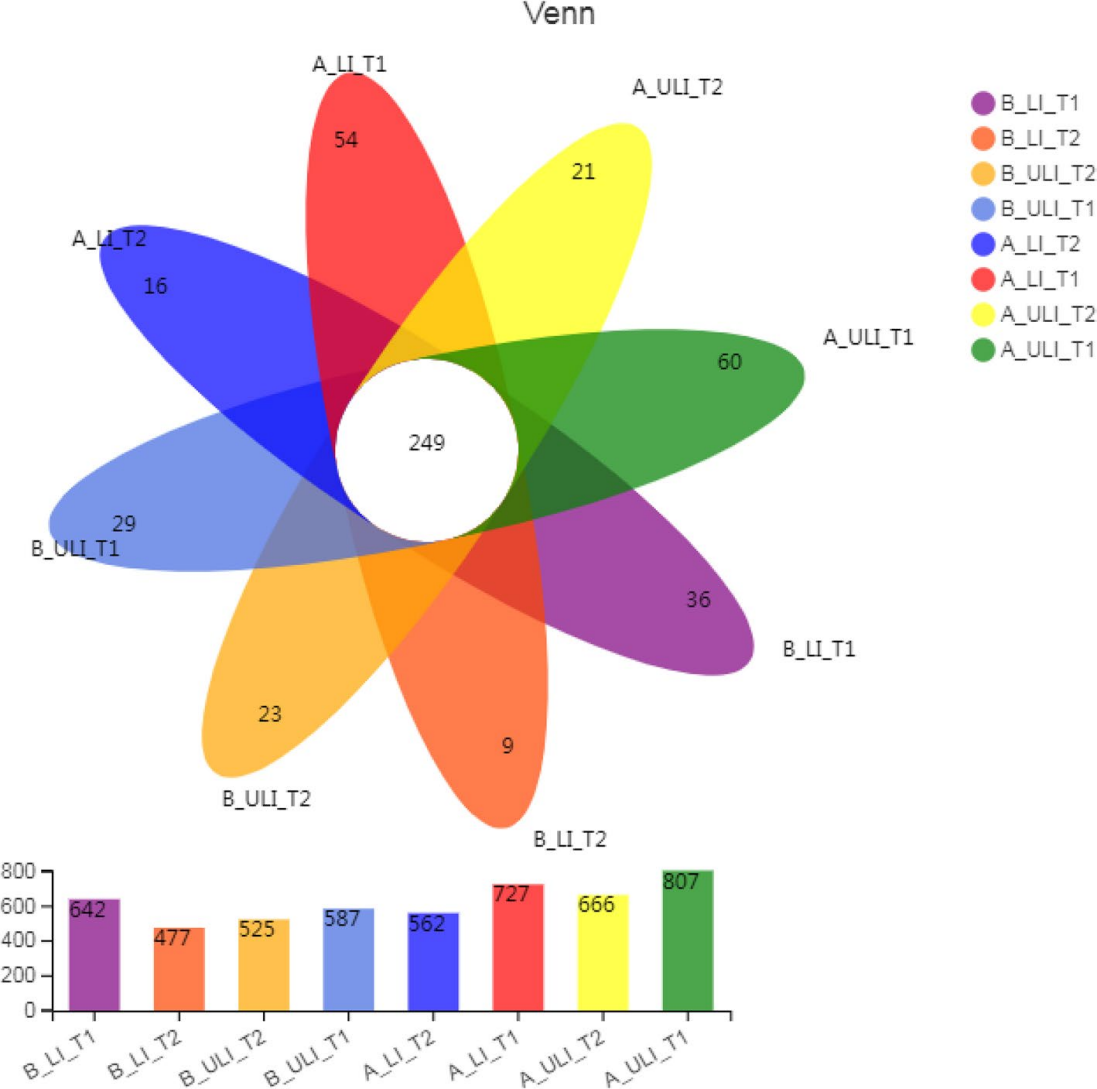
The operational taxonomic unit (OTU) of gut bacteria in various groups

A total of 5,614,215 high-quality gene sequences from 164 samples were assigned to 19 phyla, 28 classes, 69 orders, 126 families, and 384 genera in the kingdom of bacteria. The number of OTUs contained in these eight groups of samples was analysed, and the results are shown in Fig. 2. The total number of core OTUs in the eight groups was 249. It can be seen that, compared with the T1 and T2 groups, drug treatment led to a decrease in the number of OTUs in both the Mongolian and Han ethnic groups, and the number of OTUs in each group of Mongolian patients was higher than that of the Han patients (see Fig. 2).

**Fig. 3:**
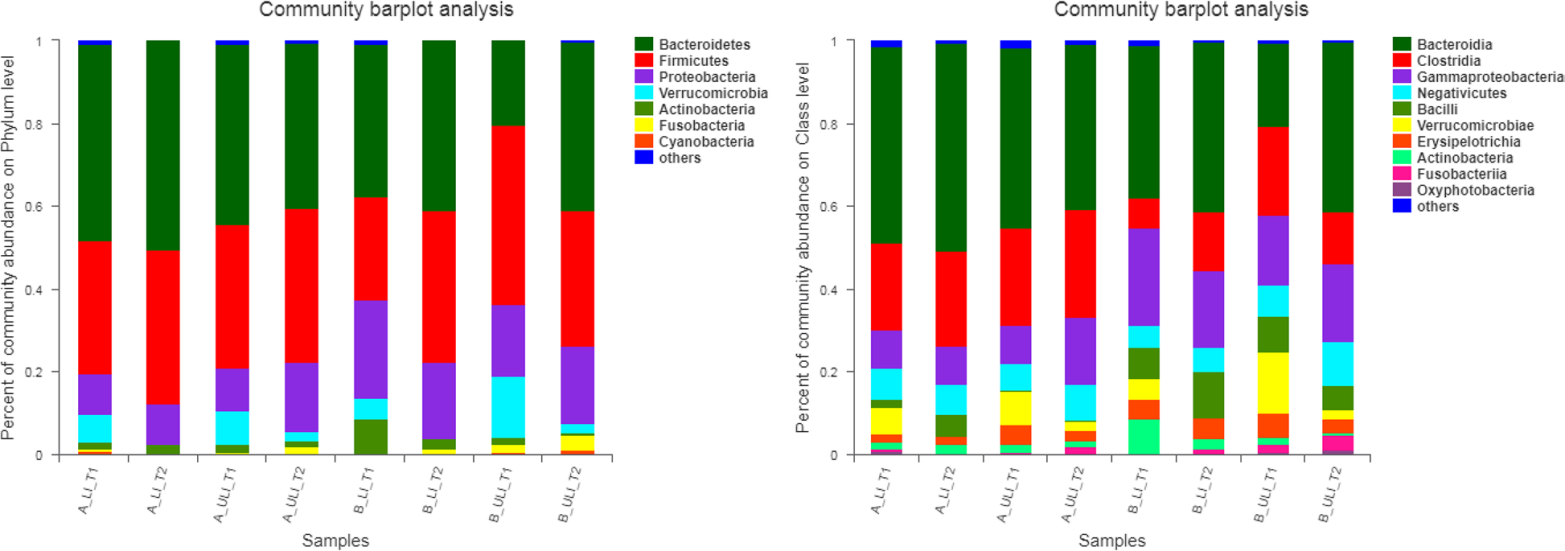
The relative abundance of species on phylum level and class level in each group. (left) phylum level. (right) class level

**Fig. 4:**
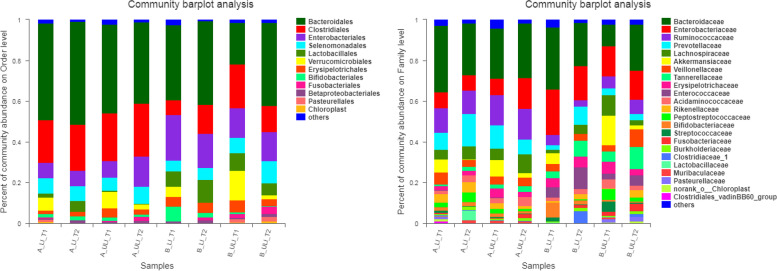
The relative abundance of species in each group on order level and family level. (left) order level. (right) family level

**Fig. 5:**
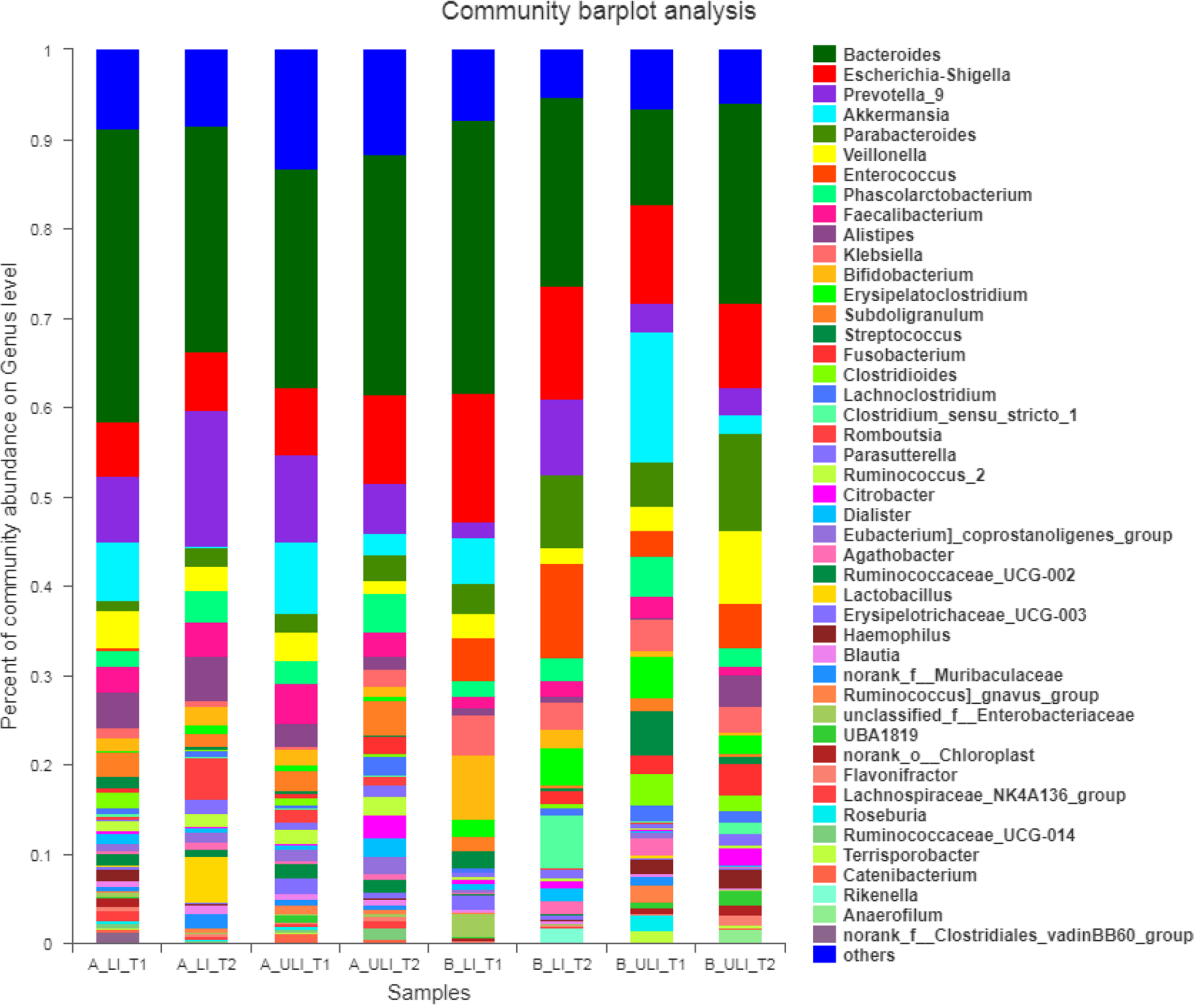
The relative abundance of species in each group on genus level

At the phylum level, the intestinal microbiota in each group of the Mongolian and Han patients mainly comprised seven phyla, while at the class level, the intestinal microbiota in each group mainly comprised 10 classes, as shown in Fig. 3. At the order level, the intestinal microbiota in each group mainly comprised 12 orders, while at the family level, the intestinal microbiota in each group mainly comprised 23 families, as shown in Fig. 4. At the genus level, the intestinal microbiota in each group consisted of 45 genera (see Fig. 5).

**Fig. 6:**
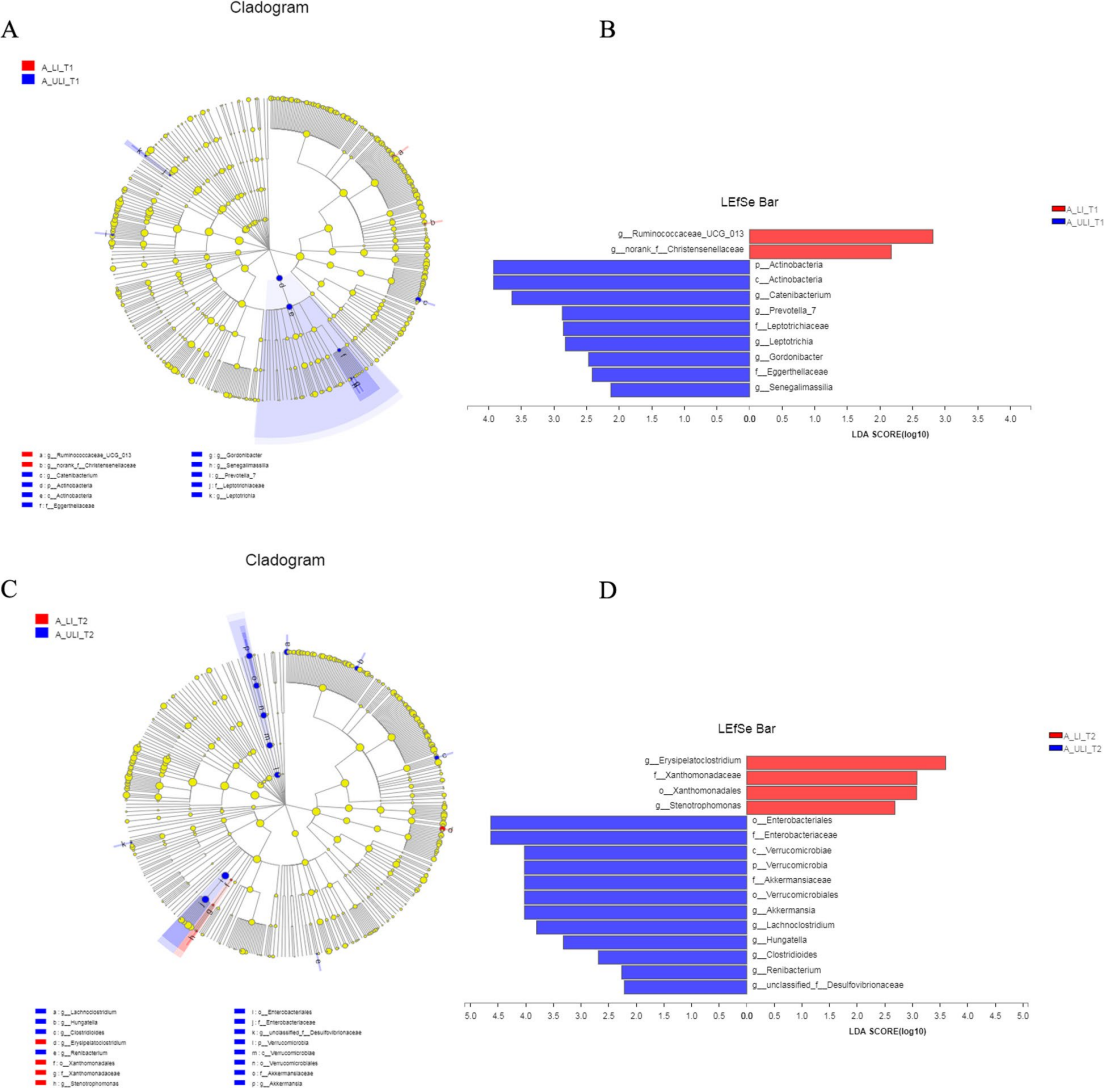
Gut bacteria differences between liver injury and non-liver injury groups of the Mongolian. **A** Cladogram in T1. **B** Linear discriminant analysis Effect Size (LEfSe) in T1. **C** Cladogram in T2. **D** Linear discriminant analysis Effect Size (LEfSe) in T2

At T1, there were significant differences in 11 taxa between the two groups of Mongolian patients (see Fig. 6). At the genus level, the relative abundances of the genera *Ruminococcaceae_UCG_013* and norank_f__*Christensenellaceae* in the A_LI_T1 group were higher, while those in the A_ULI_T1 group were of *Catenibacterium*. The relative abundances of *Prevotella_7*, *Leptotrichia*, *Gordonibacter* and *Senegalimassilia* were relatively high. At T2, there were significant differences in 16 taxa between the two groups. The genera *Erysipelatoclostridium* and *Stenotrophomonas* showed higher relative abundances in the A_LI_T2 group, while the relative abundances of the genera *Akkermansia*, *Lachnoclostridium*, *Hungatella*, *Clostridioides*, *Renibacterium* and unclassified_f__*Desulfovibrionaceae* in the A_ULI_T2 group were higher than those in the A_LI_T2 group. High. At T2, new differential bacterial genera (*Erysipelatoclostridium*, *Stenotrophomonas*, *Akkermansia*, *Lachnoclostridium*, *Hungatella*, *Clostridioides*, *Renibacterium* and unclassified_f__*Desulfovibrionaceae*) appeared in the LI group and the ULI group.

**Fig. 7:**
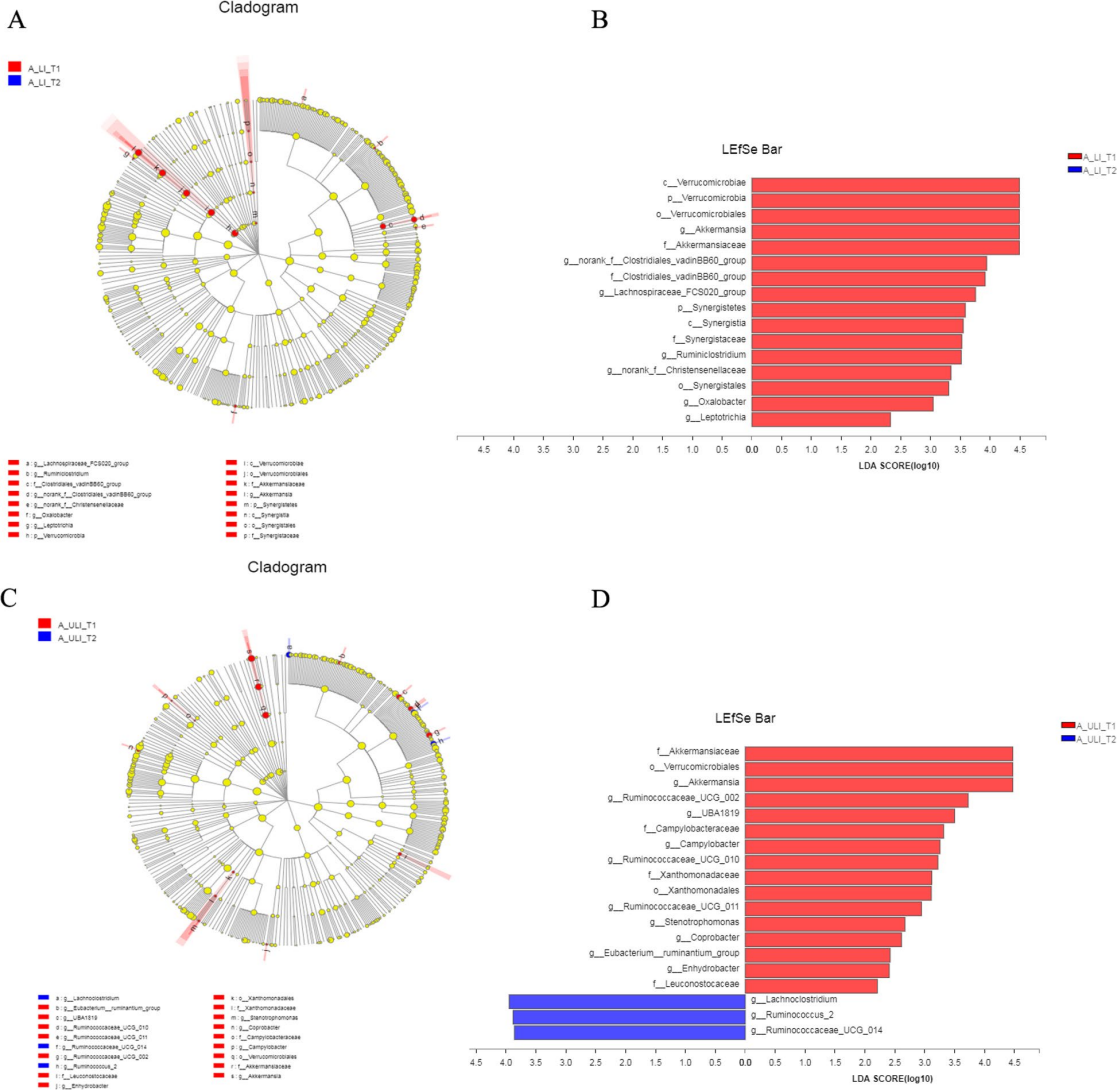
The dynamic difference of the gut bacteria liver injury and non-liver injury groups of the Mongolian from T1 to T2. **A** Cladogram in gut bacteria liver injury group. **B** Linear discriminant analysis Effect Size (LEfSe) in in gut bacteria liver injury group. **C** Cladogram in non-liver injury groups. **D** Linear discriminant analysis Effect Size (LEfSe) in non-liver injury groups

The LEfSe analysis showed that a total of 16 taxa were significantly different between T1 and T2 in the LI group (*P* < 0.05, LDA > 2) (Fig. 7), and all were significantly enriched at T1. At the genus level, the genera *Akkermansia*, norank_f__*Clostridiales_vadinBB60_group*, *Lachnospiraceae_FCS020_group*, *Ruminiclostridium*, norank_f__*Christensenellaceae*, *Oxalobacter* and *Leptotrichia* were mainly enriched. In contrast, 19 taxa differed significantly between T1 and T2 in the ULI group. At the genus level, the genera *Akkermansia*, *Ruminococcaceae_UCG_002*, *UBA1819*, *Campylobacter*, *Ruminococcaceae_UCG_010*, *Ruminococcaceae_UCG_011*, *Stenotrophomonas*, *Coprobacter*, *Eubacterium__ruminantium_group*, and *Enhydrobacter* were significantly enriched at T1. The genera *Lachnoclostridium*, *Ruminococcus_2* and *Ruminococcaceae_UCG_014* were significantly enriched at T2. The longitudinal analysis showed that three genera, *Akkermansia*, *Stenotrophomonas* and *Lachnoclostridium*, were the genera with differences in the longitudinal changes of medication in Mongolian patients as well as in A_LI_T2 and A_ULI_T2.

**Fig. 8:**
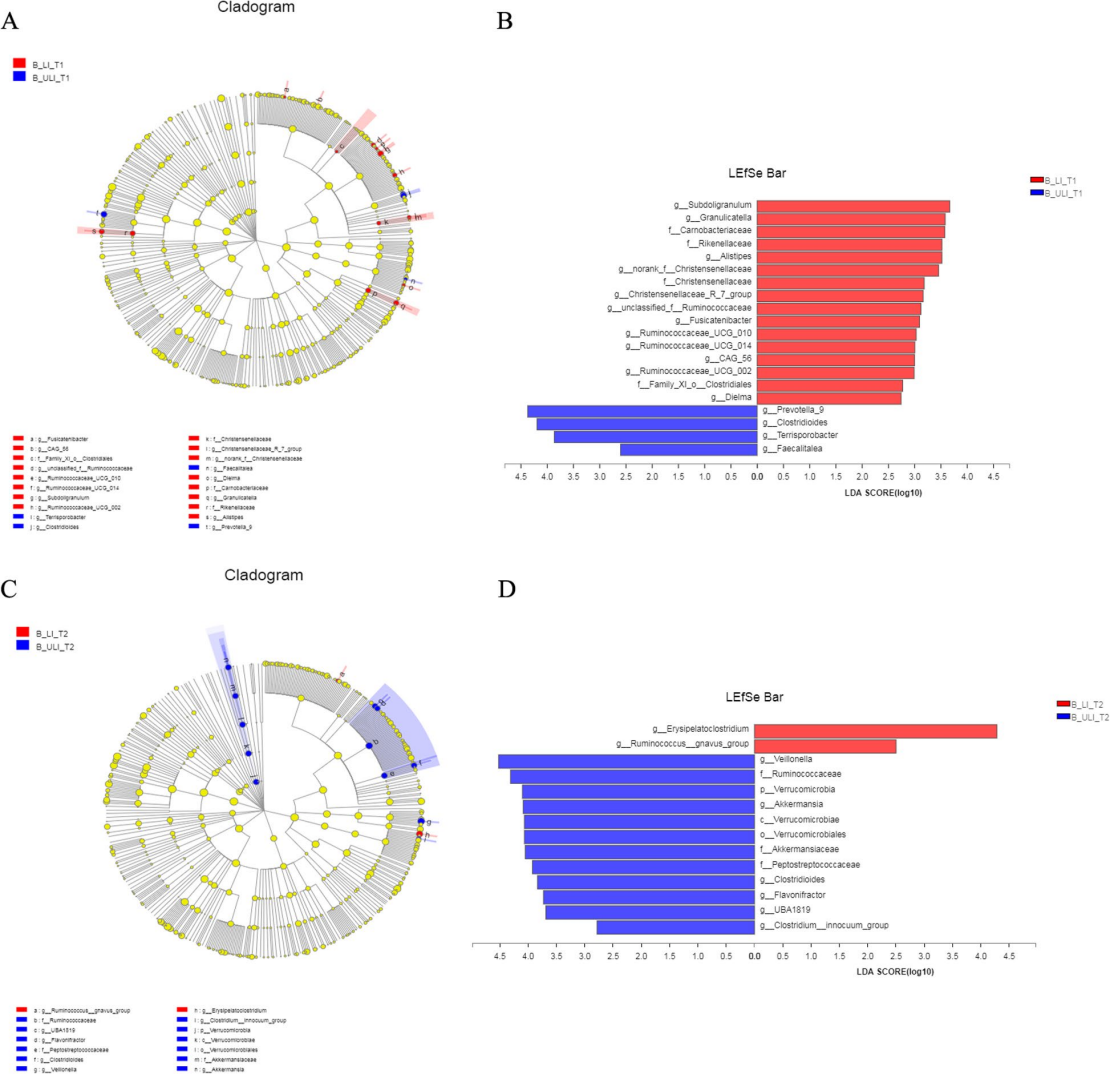
Differences in gut bacteria between liver injury and non-liver injury groups of the Han ethnic. **A** Cladogram in T1. **B** Linear discriminant analysis Effect Size (LEfSe) in T1. **C** Cladogram in T2. **D** Linear discriminant analysis Effect Size (LEfSe) in T2

Similarly, we analysed the Han patients at T1, and 20 taxa were significantly different between the two groups (Fig. 8). At the genus level, *Alistipes*, *Christensenellaceae_R_7_group*, *CAG_56*, rare small. The relative abundances of the genera *Subdoligranulum*, *Fusicatenibacter*, norank_f__*Christensenellaceae*, *Ruminococcaceae_UCG_010*, *Granulicatella*, unclassified_f__*Ruminococcaceae*, *Ruminococcaceae_UCG_002*, *Ruminococcaceae_UCG_014*, *Dielma*, and B_ULI_T1, diocese, *Terrispocaliarobacter.* The relative abundance of the genus was high. At T2, there were significant differences in 14 taxa between the two groups. The genera *Erysipelatoclostridium* and *Ruminococcus__gnavus_group* all showed higher relative abundance in the B_LI_T2 group, while the relative abundances of *Akkermansia*, *Clostridioides*, *UBA1819*, *Veillonella*, *Clostridium__innocuum_group* and *Flavonifractor* were higher in the B_ULI_T2 group. At T2, new differential bacterial genera appeared in both the LI group and the ULI group: *Erysipelatoclostridium*, *Ruminococcus__gnavus_group*, *Akkermansia*, *Clostridioides*, *UBA1819*, *Veillonella*, *Clostridium__innocuum_group* and *Flavonifractor*.

**Fig. 9:**
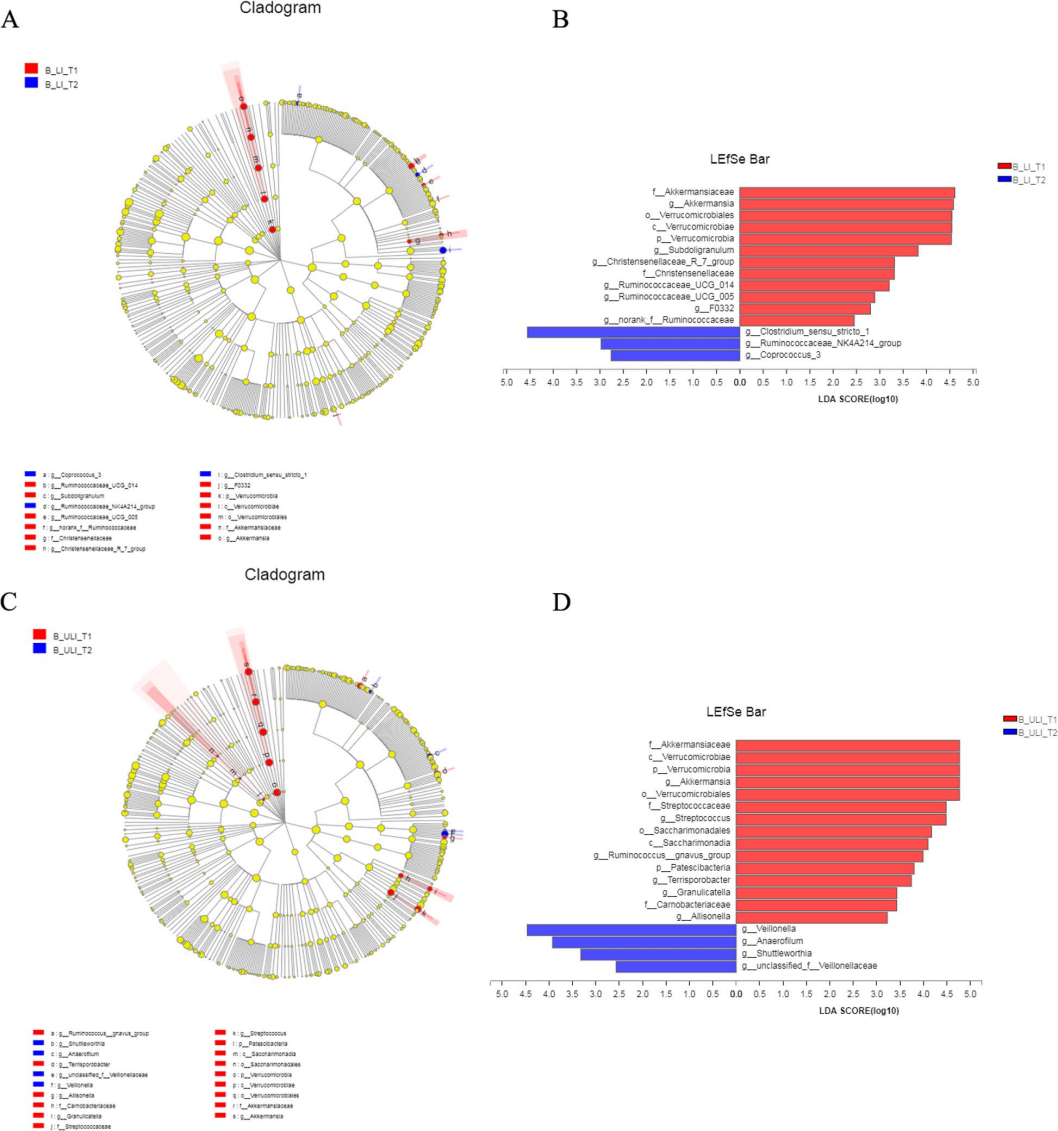
Dynamic difference of gut bacteria from T1 to T2 in liver injury and non-liver injury groups of Han nationality. **A** Cladogram in gut bacteria liver injury group. **B** Linear discriminant analysis Effect Size (LEfSe) in in gut bacteria liver injury group. **C** Cladogram in non-liver injury groups. **D** Linear discriminant analysis Effect Size (LEfSe) in non-liver injury groups

The LEfSe analysis showed that a total of 15 taxa were significantly different between T1 and T2 in the LI group (*P* < 0.05, LDA > 2) (Fig. 9).

At T1, the genera *Christensenellaceae_R_7_group*, norank_f__*Ruminococcaceae*, *F0332*, *Ruminococcaceae_UCG_014*, *Ruminococcaceae_UCG_005*, *Subdoligranulum* and *Akkermansia* were significantly enriched at the genus level. At T2, the genera *Ruminococcaceae_NK4A214_group*, *Coprococcus_3* and *Clostridium_sensu_stricto_1* were more abundant. In contrast, 19 taxa differed significantly between T1 and T2 in the ULI group. At the genus level, *Akkermansia*, *Ruminococcus__gnavus_group*, *Terrisporobacter*, *Granulicatella*, *Allisonella* and *Streptococcus* were significantly enriched at T1, while *Veillonella*, *Anaerofilum*, *Shuttleworthia* and the genus unclassified_f__*Veillonellaceae* were significantly enriched at T2. A longitudinal analysis showed that the genera *Akkermansia*, *Veillonella* and *Ruminococcus__gnavus_group* not only showed differences in longitudinal changes in Han patients after medication but also in the B_LI_T2 and B_ULI_T2 groups.

Notably, we compared the co-differential genera of the intestinal microbiota of the Mongolian and Han LI and ULI groups and found that *Akkermansia* showed consistent temporal changes in the two groups. From T1 to T2, the beneficial bacteria *Akkermansia* were significantly reduced in the Mongolian and Han ethnic groups in the LI group and the ULI group. Compared with the ULI group, the reduction in the LI group was more significant. In addition, we found that, although there was no statistical difference in longitudinal changes, the harmful genus *Erysipelatoclostridium* was higher in abundance in the Mongolian A_LI_T2 and Han B_LI_T2 groups than in those without liver injury.

### Correlation analysis of differential bacteria and serum liver function and inflammatory indexes

The correlation results showed that the Mongolian *Akkermansia* was negatively correlated with ALT, AST, ALP and TNF-α (all *P* < 0.05), and *Stenotrophomonas* was positively correlated with ALT, AST, ALP, TBIL and TNF-α (all *P* < 0.05). The genus *Akkermansia* in the Han nationality was negatively correlated with ALT, AST, ALP and IL-6 (all *P* < 0.05), and the genus *Ruminococcus_gnavus_group* was positively correlated with ALT, AST and IL-6 (all *P* < 0.05) (see Table 2 and Table 3).

**Table 2:** Correlation analysis of different gut bacteria and Serological Index in Mongolian Nationality Patients

	Akkermansia		Stenotrophomonas		Lachnoclostridium	
	*r*	*P*	*r*	*P*	*r*	*P*
ALT	−0.379	0.010	0.330	0.027	−0.115	0.452
AST	−0.364	0.014	0.321	0.032	0.067	0.664
ALP	−0.346	0.020	0.355	0.017	0.149	0.328
TBIL	−0.179	0.239	0.302	0.044	0.174	0.253
TNF-α	−0.353	0.017	0.306	0.041	0.111	0.468
IL-6	−0.184	0.226	0.183	0.229	−0.153	0.317

**Table 3:** Correlation analysis of different gut bacteria and Serological Index in Han Nationality Patients

	Akkermansia		Veillonella		Ruminococcus_gnavus_group	
	*r*	*P*	*r*	*P*	*r*	*P*
ALT	−0.364	0.027	0.020	0.907	0.456	0.005
AST	−0.377	0.021	−0.139	0.412	0.347	0.035
ALP	−0.433	0.007	0.025	0.881	0.210	0.211
TBIL	0.141	0.407	−0.129	0.447	−0.048	0.780
TNF-α	−0.154	0.363	−0.046	0.787	0.167	0.323
IL-6	−0.493	0.002	0.092	0.587	0.488	0.022

## Discussion

This study is the first to establish the relationship between the side effects of ADLI and intestinal microbiota and investigate Mongolian and Han patients with pulmonary TB and liver injury (the LI group) and no liver injury (the ULI group) taking anti-TB drugs. After identifying changes in the dynamics of their intestinal microbiota from before drug treatment (T1) to the time of liver injury (T2) after receiving anti-TB therapy, an attempt was made to identify the co-differentiated intestinal microbes associated with the occurrence of liver injury in the two groups.

The diversity of the intestinal microbiome is defined as the number and abundance distribution of different types of microorganisms colonising the intestinal tract [11]. It has been proven that the abnormal diversity of intestinal microbiota is related to human diseases. A decrease in intestinal microbiota diversity can easily induce obesity [12, 13] and diabetes [14] and may also lead to an increase in the probability of cardiovascular disease [15, 16], thereby promoting inflammatory bowel disease [17, 18], cancer, etc. [19]. Recently, a report highlighted that the intestinal microbiota is also related to the toxicity and side effects of drugs, and its flora can synthesise toxic compounds from drug components, which can damage organs [20]. The liver detoxifies drugs by adding groups to it via a glucuronidation process. However, bacterial enzymes remove the added groups and break down the drug into a toxic compound.

We compared the composition of intestinal microbial communities. We only performed a systematic analysis at the phylum level, and we observed that although the Mongolian and Han ethnic groups were different in terms of genetics, environment, diet, etc., the two ethnic groups (LI and ULI) had higher phyla, with Bacteroidetes, thick Firmicutes and Proteobacteria as the main dominant bacterial phyla of the two families. At T2, the three major bacterial phyla were still the main dominant bacterial phyla, with these three major bacterial phyla appearing in T1 and T2, respectively. There was no difference between the LI group and the ULI groups of Han patients, and there was no statistical difference in the longitudinal changes from T1 to T2 between the Mongolian and Han LI and ULI groups, indicating that people with different genetic, environmental, and dietary backgrounds are the dominant bacteria. The phylum has similarities, which are also consistent in other population studies [21]. At the same time, this also shows that anti-TB drugs have no significant effect on the three dominant bacterial phyla. In addition, we found an important phylum, Verrucomicrobia, which was different in both the Mongolian–Han LI group and the ULI group at T2, and in the dynamic comparison from T1 to T2, the Mongolian–Han ethnic group was significantly different. There were also differences, indicating that Verrucomicrobia is the phylum that responds to anti-TB drugs and may be the phylum associated with the occurrence of liver injury.

One of our objectives in this study was to compare whether the changes in the microbiota of the two ethnic groups were similar between the Mongolian and Han ethnic groups with pulmonary TB during the period from anti-TB drug treatment to liver injury. Our LEfSe data showed that the microbiota changed significantly from T1 to T2 in both the LI and ULI groups, regardless of whether they were Mongolian or Han. However, there were commonalities and personality changes in the microbiota changes from T1 to T2 in the LI and ULI groups of the two populations.

The genus that changed between the Mongolian and Han ethnic groups has its uniqueness. We hypothesise that after the Mongolian and Han ethnic groups were treated with drugs, the microorganisms of the two ethnic groups should have had more similarities, but in reality, there were not many microorganisms that mutated between the two ethnic groups. On the one hand, drug sensitivity differed between the two ethnic groups with their diverse genetic backgrounds. In addition, the flora was affected by antibiotics [22–24] and other factors, such as age [25–27], region [28–30], and diet [31–33]. This is a limitation of this study.

However, we also found that the co-differentiation of *Akkermansia* was significantly decreased from T1 to T2 in both the LI and ULI groups. In an animal study, metformin improved liver injury and colonic barrier dysfunction. In addition, metformin improved sepsis-induced liver inflammation and damage to the intestinal microbiota [34]. This means that drugs like metformin may be used to protect the liver by affecting the intestinal microbiome [35].

In this study, our main objective was to identify the microorganisms related to liver injury in Mongolian and Han populations. The genera that are caused by drugs from T1 to T2 may be related to the occurrence of liver injury due to adverse drug reactions. Therefore, we further compared and analysed the differential bacteria in the LI and ULI groups. Finally, it was caused by drugs, and it was different from the Mongolian liver injury. The genera associated with the injury were *Akkermansia*, *Stenotrophomonas* and *Lachnoclostridium*, and the genera associated with liver injury in the Han population were *Akkermansia*, *Veillonella* and *Ruminococcus__gnavus_group*.

Additionally, we conducted a correlation analysis of the abovementioned bacteria that may be related to the occurrence of liver injury in the Mongolian and Han ethnic groups using serum liver function indexes ALT, AST, ALP and TBIL, and serum inflammation markers TNF-α and IL-6. There were negative correlations with ALT, AST, ALP and TNF-α. *Stenotrophomonas* had positive correlations with ALT, AST, ALP, TBIL and TNF-α, while *Lachnoclostridium* did not have physical clinical indicators. In the Han population, the genus *Akkermansia* was negatively correlated with ALT, AST, ALP and IL-6, and the genus *Ruminococcus_gnavus_group* was positively correlated with ALT, AST and IL-6; however, the genus *Veillonella* did not correlate with these clinical indicators. The results of the correlation analysis suggested that *Akkermansia* and *Stenotrophomonas* were related to the occurrence of liver injury in the Mongolian population, while *Akkermansia* and *Ruminococcus__gnavus_group* were related to the occurrence of liver injury in the Han population. *Akkermansia* was related to the occurrence of liver injury in both the Mongolian and Han ethnic groups and belonged to the phylum Verrucomicrobia, which was consistent with the differences at the phylum level. These findings suggest that anti-TB drug treatment increases the propensity of patients with pulmonary TB to develop a liver injury, which may be related to drugs targeting specific altered bacteria in the gut. In addition, it also suggested that *Akkermansia* may be related to the occurrence of liver injury, which is worthy of further study.

Some limitations of this study should be mentioned. Intestinal microbiota can be influenced by multiple factors. Although patients were not allowed to use antibiotics, probiotics, and prebiotics in our study, participants were not given a standardised diet during treatment. Although this study assessed the effects of anti-TB drug treatment on the gut ecosystem, patients were assessed at only two time points. Therefore, our study cannot explain whether changes in microbiota are a cause or a consequence of liver injury. Longitudinal designs in patients with or without liver injury are needed to elucidate the causal relationship between ADLI and intestinal microbiota.

## Conclusion

Anti-TB drugs do not alter the structure of intestinal microbiota. The intestinal bacteria *Akkermansia* and *Stenotrophomonas* altered by anti-TB drugs were associated with the occurrence of liver injury in Mongolian patients, and the altered genera *Akkermansia* and *Ruminococcus gnavus_group* were associated with the occurrence of liver injury in Han patients. The genus *Akkermansia* was related to the occurrence of ADLI in both the Mongolian and Han ethnic groups.

## Data Availability

All data generated or analyzed during this study are included in this published article.
